# Exosomes derived from senescent skeletal muscle cells aggravate nucleus pulposus cell metabolic dysregulation via p38MAPK pathway for promoting intervertebral disc degeneration

**DOI:** 10.1186/s12891-026-09816-8

**Published:** 2026-04-16

**Authors:** Xiaowei Ma, Weiqi Zhang, Han Yin, Dazhuang Miao, Xianda Gao, Chunxu Fu, Wei Chen, Zhiyong Hou, Qi Zhang, Yingze Zhang, Di Zhang

**Affiliations:** 1 Department of Orthopaedic Surgery, Hebei Medical University Third Hospital, Shijiazhuang, Hebei Province 050051 China; 2 NHC Key Laboratory of Intelligent Orthopaedic Equipment, Hebei Medical University Third Hospital, Shijiazhuang, Hebei Province 050051 China; 3 Orthopaedic Research Institution of Hebei Province, Hebei Medical University Third Hospital, Shijiazhuang, Hebei Province 050051 China; 4 Department of Orthopaedic, Union Hospital, Tongji Medical College, Huazhong University of Science and Technology, Wuhan, Hubei Province 430022 China; 5 School of Medicine, Nankai University, Tianjin, 300071 China; 6 Chinese Academy of Engineering, Beijing, 100088 China

**Keywords:** Intervertebral disc degeneration, Senescent skeletal muscle cells, Exosomes, Nucleus pulposus cells, p38MAPK pathway

## Abstract

**Background:**

Intervertebral disc degeneration (IVDD) is the primary pathological driver of chronic low back pain. While the mechanical role of skeletal muscle in spinal stability is well-established, its paracrine influence on IVDD—specifically via exosomes—remains poorly understood. This study investigated whether senescent skeletal muscle cell-derived exosomes (sSkM-Exos) aggravate metabolic dysregulation in nucleus pulposus cells (NPCs) and explored the underlying molecular mechanisms.

**Methods:**

Senescence was induced in skeletal muscle cells (SkMCs) using hydrogen peroxide (H₂O₂), and sSkM-Exos were isolated via differential centrifugation and characterized. The internalization of sSkM-Exos by NPCs was observed. In vitro, the effects of sSkM-Exos on NPCs proliferation, senescence, apoptosis, and extracellular matrix (ECM) metabolism (COL2, ACAN, MMP13, and ADAMTS5) were evaluated. The involvement of the p38MAPK pathway was assessed using the inhibitor SB203580. *In vivo*, the impact of sSkM-Exos was validated using a rat IVDD model, monitored by disc height, MRI T2-weighted signaling, and histological analysis.

**Results:**

We induced senescent skeletal muscle cells (SkMCs) with hydrogen peroxide (H₂O₂), extracted and identified sSkM-Exos via differential centrifugation. Our findings demonstrate that sSkM-Exos can be internalized by NPCs. In vitro, sSkM-Exos enhanced H₂O₂-induced NPC proliferation inhibition, senescence, apoptosis, and extracellular matrix (ECM) imbalance (downregulated COL2/ACAN, upregulated MMP13/ADAMTS5). Mechanistically, these effects were associated with p38MAPK activation; the p38MAPK inhibitor SB203580 partially reversed these impairments. *In vivo*, local sSkM-Exos injection was observed to exacerbate disc height loss and histological degeneration in a rat IVDD model.

**Conclusions:**

In conclusion, sSkM-Exos were found to contribute to NPC senescence and ECM imbalance, likely through the activation of the p38MAPK pathway. These findings propose a cross-tissue regulatory network that supplements the traditional biomechanical model with a molecular perspective under experimental conditions. This study offers a new dimension to our understanding of IVDD pathogenesis and suggests that targeting sSkM-Exos or the p38MAPK pathway may hold promise as a therapeutic strategy to mitigate IVDD.

**Supplementary Information:**

The online version contains supplementary material available at 10.1186/s12891-026-09816-8.

## Background

Low back pain (LBP) is a highly prevalent public health issue worldwide, with a lifetime prevalence rate ranging from 70% to 85%, significantly impairing patients’ quality of life and contributing to a substantial socioeconomic burden on global healthcare systems [1]. IVDD represents the primary pathological cause underlying chronic LBP and neck pain. Its incidence has been steadily rising amid the global aging population, emerging as a major challenge confronting the fields of geriatrics and orthopedics. Epidemiological data indicate that approximately 80% of adults will experience low back pain of varying severity during their lifetime, among which over 60% of cases are directly associated with IVDD [2, 3].

With in-depth exploration of the pathological progression of IVDD, researchers have gradually clarified that its core pathological mechanism involves multi-dimensional disorders, including NPCs dysfunction, imbalanced degradation of ECM, overactivation of oxidative stress, and formation of a chronic inflammatory microenvironment [4–9]. Currently, clinical treatments for advanced IVDD (such as medication, minimally invasive intervention, and surgical fusion) can only relieve symptoms or reconstruct mechanical structures, lacking biological approaches to block or reverse the degeneration [7–10]. The core reason is that the initiating factors and regulatory mechanisms remain unclear. As the core functional unit of the intervertebral disc, NPCs are key maintainers of ECM homeostasis, and their functional abnormalities (decreased proliferation, activated senescence and apoptosis, and imbalanced synthesis and degradation of ECM) are the core of the initiation and progression of IVDD: the synthesis of molecules such as type Ⅱ collagen (COL2) and aggrecan (ACAN) is down-regulated, while matrix degrading enzymes (matrix metalloproteinase 13 [MMP13], a disintegrin and metalloproteinase with thrombospondin motifs 5 [ADAMTS5]) are abnormally highly expressed, directly leading to dehydration and collapse of the intervertebral disc [11, 12].

Previous studies have largely centered on the mechanisms underlying metabolic dysregulation in local intervertebral disc cells in the context of mechanical loading, genetic susceptibility, nutritional deprivation, and inflammatory microenvironments [12–14]. In recent years, the proposal of the “cell-nonautonomous senescence” theory has provided a novel perspective for deciphering the intercellular regulatory mechanisms of degenerative diseases: senescent cells within the tissue microenvironment can “infect” adjacent healthy cells via paracrine signals, thereby accelerating organ functional decline [15, 16]. As the most critical mechanical support and metabolic interaction counterpart of the intervertebral disc, whether the senescent phenotype of paravertebral muscle (PM) mediates NPCs senescence through secretory signals and ultimately drives IVDD progression remains to be supported by direct biological evidence.

Existing evidence has demonstrated that PM atrophy and fat infiltration can promote annulus fibrosus rupture and nucleus pulposus dehydration by impairing vertebral stability and altering the spinal mechanical environment (e.g., increasing intradiscal pressure and enhancing shear stress) — thus forming the mechanical regulatory axis of the “muscle-disc” interaction [17, 18]. However, beyond biomechanical factors, the structural foundation for the cross-tissue communication between skeletal muscle and the intervertebral disc lies in the specialized vascular and nutritional pathways of the spine. Although the nucleus pulposus is the largest avascular tissue in the human body, it remains highly sensitive to systemic and local paracrine signals. The paravertebral muscles are supported by an extensive microvascular network that serves as a reservoir for muscle-derived secretomes. These secretomes can be transported via the capillaries of the subchondral bone into the vertebral endplate. As the primary nutritional route for the IVDD, the porous structure of the cartilaginous endplate allows for the passive diffusion and active transport of molecules and vesicles from the systemic circulation into the disc matrix [19]. This microvascular-endplate pathway facilitates a direct endocrine or paracrine link, enabling skeletal muscle-derived factors to bypass mechanical barriers and directly interact with NPCs.

Exosomes, which are natural extracellular vesicles with a diameter of 30 to 150 nm, mediate intercellular long-distance communication through the transport of proteins, lipids, and nucleic acids, and have become a hot topic in the research on the pathogenesis and IVDD [20]. Currently, most studies focus on the protective effects of mesenchymal stem cell-derived exosomes, which have been confirmed to alleviate IVDD by promoting the proliferation of NPCs, inhibiting cellular senescence, and restoring the balance of ECM metabolism [21, 22]. In contrast, senescent cell-derived exosomes—core carriers of the senescence-associated secretory phenotype (SASP)—have been shown to mediate “senescence transmission” and exacerbate various degenerative diseases, yet their pathological role in IVDD remains poorly investigated systematically [23]. As the largest tissue in the human body, skeletal muscle not only serves as a core contributor to spinal mechanical stability but also regulates the surrounding microenvironment via paracrine signaling. Numerous clinical observations have shown that skeletal muscle aging (sarcopenia) and IVDD co-occur in an age-dependent manner, suggesting a deep pathological association between the two [24]. However, whether sSkM-Exos regulate the function of NPCs and the progression of IVDD, including their uptake by NPCs and their effects on proliferation, senescence, and ECM metabolism, remains unclear. Given the highly synchronous timing of muscle aging and IVDD, we propose the core hypothesis that senescent skeletal muscle cells release exosomes rich in pro-senescence signals, which can accelerate the senescence process of NPCs and thereby exacerbate IVDD.

The mitogen-activated protein kinase (MAPK) pathway—particularly the p38MAPK subtype—acts as a pivotal signaling axis that regulates cell proliferation, senescence, apoptosis, and ECM metabolism [25, 26]. Accumulating evidence has demonstrated that aberrant activation of the p38MAPK pathway is tightly linked to the pathological progression of IVDD, and targeted inhibition of this pathway can effectively ameliorate NPCs dysfunction and delay the degenerative process of intervertebral discs [27]. However, whether sSkM-Exos regulate NPC function and IVDD progression by activating the p38MAPK pathway has not yet been experimentally validated.

This study aims to clarify the pathological role and molecular mechanism of sSkM-Exos in IVDD: specifically, to characterize its biological properties and interaction with NPCs, explore its regulatory effects on NPC functions in vitro, verify its impact on IVDD progression *in vivo*, and elucidate the underlying mechanism as well as validate the key signaling pathways. This study reveals the pathogenesis of IVDD from a novel perspective of “PM aging–exosomes–NPCs aging”, laying a solid foundation for the development of innovative biological intervention strategies and therapeutic targets targeting the “muscle–intervertebral disc” crosstalk.

## Materials and methods

### Experimental animals

Specific pathogen-free male Sprague-Dawley rats, including newborn (1–3 days old) and 8-week-old individuals, were purchased from Hebei Provincial Center for Experimental Animals (Shijiazhuang, China). The animals were housed in SPF-grade facilities with controlled ambient temperature (22–25℃), relative humidity (50%–60%), and a 12 h light–12 h dark circadian cycle. Standard chow and water were provided ad libitum throughout the study. Specifically, at the end of the experiment, all rats were euthanized by an intraperitoneal (i.p.) injection of an overdose of sodium pentobarbital at a dose of 150 mg/kg. Before administration, the rats were confirmed to be in a state of deep anesthesia. Cessation of heartbeat and loss of the pedal withdrawal reflex were used as criteria to confirm death. This method was chosen to minimize animal distress. All animal experimental protocols were reviewed and approved by the Animal Experimental Ethics Committee of the Third Hospital of Hebei Medical University, and strictly conducted in accordance with the 3R principles.

### Cell culture

Primary rat skeletal muscle cells (SkMCs) were isolated from the hindlimb skeletal muscles of newborn SD rats [28]. Specifically, newborn SD rats were first sterilized with 75% ethanol solution. The hindlimb skeletal muscle tissues were then excised, with fascia and adipose tissues carefully removed. The cleaned muscles were minced into 1-mm³ fragments, which were digested with 0.2% type Ⅱ collagenase at 37℃ for 4 h, followed by a subsequent digestion with 0.25% trypsin-EDTA at 37℃ for 30 min. Digestion was terminated by adding complete growth medium, and the cell suspension was filtered through a sterile 200-mesh cell strainer. Cells were collected by centrifugation, adjusted to a density of 5 × 10⁵ cells/mL, and seeded into sterile culture dishes in DMEM/F12 medium supplemented with 10% FBS and 1% penicillin-streptomycin. Cultivation was performed in a humidified incubator at 37℃ with 5% CO₂, and the medium was refreshed every other day. Cells at Passage 3 were selected for subsequent experiments.

Primary rat NPCs were isolated from the nucleus pulposus tissues of caudal Co4-Co6 intervertebral discs of 8-week-old SD rats. After the 8-week-old SD rats were humanely euthanized, the caudal Co4-Co6 intervertebral discs were aseptically isolated, and the nucleus pulposus tissues were dissected and minced. The tissue fragments were digested with a mixture of 0.25% trypsin-EDTA and type Ⅱ collagenase. Subsequent culture was carried out as per the SkMC protocol, and cells at Passages 2–3 were used for experiments.

### Cell viability assay

The viability of NPCs following different treatments was assessed utilizing the Cell Counting Kit-8 (CCK-8) assay. Specifically, NPCs were seeded in 96-well plates at a density of 5 × 10³ cells/well, subsequently grouped according to the predefined experimental intervention regimens, and cultured for 24 h. Following the 24-hour incubation period, 10 µL of CCK-8 working solution was added to each well. The cells were then incubated in a humidified 37 °C incubator with 5% CO₂ for 4 h. Finally, absorbance values at 450 nm were detected using a microplate reader.

### Isolation and identification of exosomes

Culture supernatants were collected from normal skeletal muscle cells and senescent skeletal muscle cells, and exosomes were isolated using a differential centrifugation protocol [29]. Specifically, the supernatants were centrifuged at 300×g for 10 min at 4℃ to remove cell debris, subsequently centrifuged at 2000×g for 20 min at 4℃ to eliminate apoptotic bodies, and then centrifuged at 10,000×g for 30 min at 4℃ to remove microvesicles. Finally, exosome pellets were harvested by ultracentrifugation at 120,000×g for 70 min at 4℃, resuspended in phosphate-buffered saline (PBS), and stored at -80℃ until use. Morphological characterization by transmission electron microscopy (TEM): 20 µL of exosome resuspension was spotted onto a copper grid, negatively stained with phosphotungstic acid, and air-dried naturally prior to TEM observation and imaging. Nanoparticle tracking analysis (NTA) for particle size distribution: the exosome resuspension was diluted to an optimal concentration, and the particle size and concentration were determined using an NTA instrument. Protein concentration quantification by the BCA method: the assay was conducted in strict accordance with the manufacturer’s kit instructions, and a standard curve was plotted to calculate the exosome protein concentration. Exosomes (1 µg/µL) were labeled with the PKH-26 fluorescent dye, incubated at 37℃ for 30 min, and washed three times with PBS to remove unbound free dye. NPCs were seeded in confocal culture dishes at a density of 5 × 10⁴ cells/well and allowed to adhere and culture for 24 h. The labeled exosomes were then added to the dishes, followed by co-incubation for 6 h and 12 h, respectively. After washing with PBS, cell nuclei were counterstained with DAPI, and the distribution of fluorescent signals was visualized using a confocal laser scanning microscope.

### Real-time quantitative polymerase chain reaction (RT-qPCR)

Total RNA was isolated using TRIzol reagent (Invitrogen, USA). Complementary DNA (cDNA) was synthesized using a cDNA Synthesis Kit (Vazyme Biotech Co., Ltd., China). cDNA amplification was carried out using an RT-qPCR Kit, and specific primer sequences are provided in Supplementary Table S1. Glyceraldehyde-3-phosphate dehydrogenase (GAPDH) was used as the reference gene for error calibration. The relative mRNA expression levels were determined using the 2^−ΔΔCt^ method.

### Western blot (WB) analysis

Briefly, NPCs were lysed with lysis buffer on ice for 30 min. Subsequently, the protein concentration of the lysates was quantified using a BCA Protein Quantification Kit. Proteins were separated via SDS-PAGE electrophoresis and subsequently transferred onto polyvinylidene fluoride (PVDF) membranes. The membranes were blocked with 5% bovine serum albumin (BSA) at ambient temperature for 1 h, followed by incubation with primary antibodies at 4℃ overnight (primary antibody information is listed in Supplementary Table S2). On the following day, after incubation with appropriate secondary antibodies at ambient temperature, chemiluminescent detection was conducted to visualize the target protein bands. GAPDH was used as the internal control to normalize the protein expression levels.

### EdU cell proliferation staining

Cell proliferation was evaluated using the EdU In Vitro Detection Kit in strict accordance with the manufacturer’s protocols. Specifically, EdU reagent was added to the treated nucleus pulposus cells (NPCs) for co-incubation at 37℃ with 5% CO₂, after which the cells were subjected to sequential fixation, permeabilization, and staining procedures. Finally, fluorescent signals were visualized under a fluorescence microscope, and images were acquired for subsequent analysis.

### Cell migration assay

Wound Healing Assay: NPCs were seeded in 6-well plates and cultured to 70–80% confluency. Uniform linear scratches were created across the cell monolayer using a sterile 200 µL pipette tip. After being washed twice with PBS to remove detached cells, each well was supplemented with medium containing different treatments. Images of the scratch regions were captured at 0 h, 18 h, and 36 h respectively, and the wound healing rate was quantified using ImageJ software.

Transwell Assay: Serum-free medium containing 1 × 10⁵ NPCs was added to the upper chamber of 8 μm pore size Transwell inserts (Corning, USA), while the lower chamber was filled with medium supplemented with different treatments. After being incubated at 37℃ with 5% CO₂ for 18–36 h, non-migrated cells on the upper surface of the inserts were gently wiped off using a cotton swab. Migrated cells adhering to the lower surface were fixed with 4% paraformaldehyde at room temperature for 15 min and stained with 0.1% crystal violet solution for 20 min. Stained cells were observed under a light microscope and quantified by counting five random fields per insert.

### Cell apoptosis assay

According to the manufacturer’s instructions, the apoptosis of NPCs was detected using the Annexin V-FITC/PI Apoptosis Detection Kit (Beyotime Biotechnology Co., Ltd., China). Cell samples were collected, washed three times with PBS, and resuspended in binding buffer; 5 µL of Annexin V-FITC and 5 µL of PI were added to the cell suspension and incubated in the dark for 15 min; finally, the samples were analyzed using a flow cytometer.

### SA-β-galactosidase staining (SA-β-gal staining)

After cells were subjected to different intervention treatments, an appropriate volume of SA-β-galactosidase staining fixative was added to cover the cell monolayer, followed by fixation at room temperature for 15 min. Subsequently, the cells were rinsed twice with PBS to remove residual fixative. An appropriate amount of staining solution (Beyotime Biotechnology Co., Ltd., Shanghai, China) was then added to each culture well, and the cells were incubated at 37℃ in a CO₂-free incubator for 24 h. Finally, stained cells were visualized and imaged using a high-resolution light microscope for subsequent analysis.

### Immunocytochemistry (ICC) staining

NPCs were seeded in 24-well plates at a density of 5 × 10⁴ cells/well and subjected to different intervention conditions. After the intervention, the cells were sequentially processed as follows: washed with PBS three times, fixed with 4% paraformaldehyde at room temperature for 15 min, and permeabilized with 0.1% Triton X-100 for 15 min. Subsequently, non-specific binding sites were blocked with a rapid blocking buffer at room temperature for 30 min, followed by incubation with appropriate primary antibodies at 4℃ overnight. On the next day, the cells were rinsed three times with PBS to remove unbound primary antibodies, then incubated with corresponding fluorescent secondary antibodies at room temperature for 1 h in the dark. Finally, the cells were stained with DAPI for 15 min to label cell nuclei, and rinsed again with PBS before imaging.

### Safranin O staining

After culturing the NPCs inoculated in 12-well plates under different conditions for 24 h, they were fixed with 4% paraformaldehyde. Then, 300 µL of safranin O staining solution was added for 30 min. After discarding the staining solution, the cells were observed under a microscope.

### Alcian blue staining

As mentioned above, fix with 4% paraformaldehyde solution for 30 min, then aspirate the paraformaldehyde solution. Add 1 mL of Alcian Blue staining solution and stain for 30 min. Aspirate and discard the staining solution, then observe under a microscope.

### RNA sequencing and bioinformatics analysis

Total RNA was isolated and purified using TRIzol reagent (Invitrogen, USA) following the manufacturer’s standard operating procedures. The purified total RNA was then sent to OE-Bio for mRNA sequencing. Library construction was performed using the VAHTS Universal V10 RNA-seq Library Preparation Kit (Pre-mixed), with operations strictly following the manufacturer’s protocols. Sequencing was conducted on the Illumina Novaseq 6000 platform, generating 150 bp paired-end reads. Differentially expressed genes (DEGs) were screened using the criteria of Q value < 0.05 and fold change > 2 or < 0.5. R software (Version 3.2.0) was employed to perform Gene Ontology (GO) functional enrichment analysis and Kyoto Encyclopedia of Genes and Genomes (KEGG) pathway enrichment analysis on DEGs. Significantly enriched terms were identified, and bar charts were generated using the same software. Additionally, Gene Set Enrichment Analysis (GSEA) was performed using GSEA software.

### Animal model

Specifically, the rats were anesthetized via an intraperitoneal injection of pentobarbital sodium at a dose of 45 mg/kg body weight. The depth of anesthesia was carefully monitored by observing the corneal reflex, muscle relaxation, and response to toe-pinch (pain stimulus). Surgical procedures were initiated only after ensuring the animal had reached a stable and adequate plane of anesthesia. During the surgery, supplemental doses were administered, if necessary, based on the animal’s physiological state, to maintain continuous and effective anesthesia. To establish a rat IVDD model via puncture, the target caudal vertebral segment was first palpated to roughly localize its position. After disinfection of the surgical site, a 21G puncture needle was held perpendicular to the skin surface and parallel to the vertebral endplates, then slowly advanced into the center of the target intervertebral disc space, with a depth of approximately 5 mm. The needle was rotated 360 degrees, maintained in that position for 30 s to ensure sufficient injury, and then gently withdrawn. Local drug delivery was performed once a week post-modeling. After each local injection, the animals were returned to their individual cages and allowed unrestricted activity ad libitum.

### Paraffin section immunofluorescence staining

Paraffin sections were first subjected to standard deparaffinization and rehydration procedures. Afterward, the sections were blocked with 10% goat serum at room temperature for 30 min to block non-specific antigen binding sites. Primary antibodies (diluted 1:200 in antibody dilution buffer) were then added, and the sections were incubated at 4℃ overnight in a humidified chamber. On the next day, after three washes with PBS, corresponding fluorescent secondary antibodies (Zhongshan Jinqiao Biotechnology Co., Ltd., China) were added for incubation at room temperature for 30 min in the dark. Finally, the sections were stained with DAPI for 10 min to label cell nuclei, followed by gentle rinsing with PBS to remove excess dye for subsequent fluorescence microscopy observation.

### Hematoxylin-eosin (HE) staining and safranin O-fast green staining

Sections were baked at 70℃ for 2 h to enhance tissue adhesion to glass slides. After undergoing standard deparaffinization and rehydration procedures (to remove paraffin and restore tissue hydration), the sections were subjected to HE staining and Safranin O-Fast Green staining, respectively, following standard protocols. Subsequent to staining, the sections were dehydrated through a graded ethanol series (95% ethanol twice followed by 100% ethanol twice) to remove residual water. After dehydration, the sections were mounted with neutral balsam and allowed to dry completely. Finally, the intervertebral disc tissues were observed under a light microscope, and representative images were captured to assess the degree of IVDD injury, including structural integrity and ECM changes.

### X-ray and MRI examinations

Preoperatively and at 4 and 8 weeks postoperatively, a veterinary-specific X-ray machine was used to acquire X-ray images of the rat tail. The disc height index (DHI) was calculated from the obtained images following protocols established in previous studies [21], as shown in Figure S1 (Supporting Information). Additionally, T2-weighted MRI images of the rat tail were acquired using an Achieva 3.0T MRI system (Philips, Amsterdam, the Netherlands). The degree of IVDD was measured and evaluated by an assessment team comprising three or more attending orthopedic surgeons, who were blinded to the experimental groups and followed preset standardized criteria.

### Statistical analysis

GraphPad Prism 10 software (GraphPad Software, La Jolla, California, USA) was employed for statistical analysis and data visualization. All experimental data were presented as mean ± standard deviation (mean ± SD). Unpaired Student’s t-test was applied for comparisons between two independent groups, while one-way analysis of variance (one-way ANOVA) followed by Tukey’s post-hoc test was used for multiple group comparisons. Statistical significance was defined as *p < 0.05, **p < 0.01, ***p < 0.001, and ****p < 0.0001. “ns” indicates no significant differences.

## Results

### Extraction and characterization of SkMCs-derived exosome

**Fig. 1:**
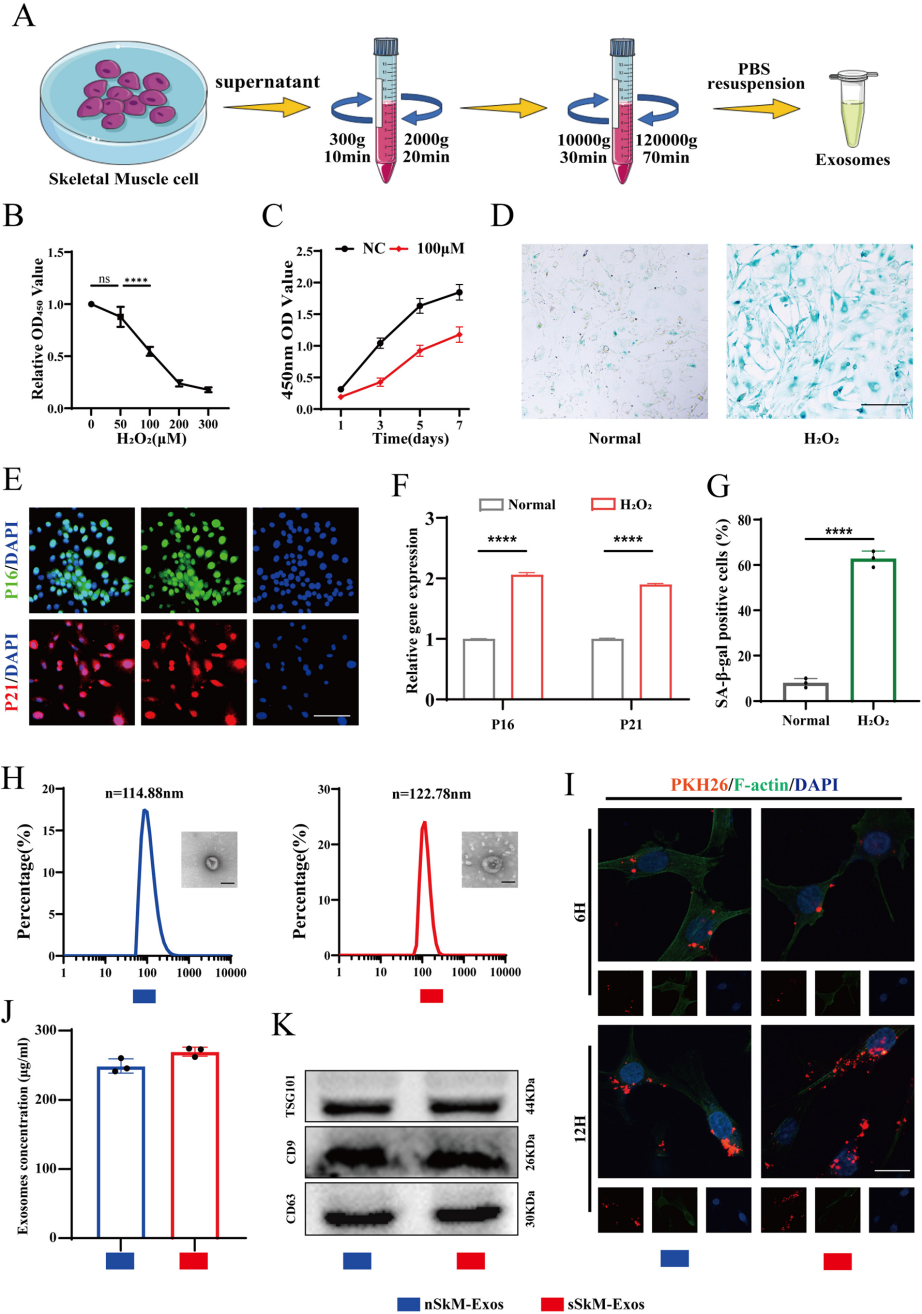
Establishment and validation of senescent SkMCs model and preparation and characterization of SkMCs-derived exosomes. **A**. Schematic diagram of exosome isolation. **B.** Cell viability quantification of SkMCs treated with gradient concentrations of H₂O₂. **C**. Cell viability assay of SkMCs after serial continuous treatment with the optimized H₂O₂ concentration. **D**, **G**. SA-β-galactosidase staining (representative images) and quantitative analysis results of SkMCs. Scale bar, 100 μm. **E**. Representative fluorescent images of senescence-associated markers in SkMCs following H₂O₂-induced senescence. Scale bar, 50 μm. **F**. Relative gene expression levels of senescence markers in SkMCs after H₂O₂ treatment. **H**. NTA size distribution results and representative TEM morphological images of nSkM-Exos and sSkM-Exos. Scale bar, 200 nm. **I**. Representative fluorescent images of PKH-26-labeled exosomes co-cultured with NPCs for 6 h and 12 h. Scale bar, 20 μm. **J**. Quantification results of exosome protein concentration. **K**. Quantitative analysis of surface marker proteins (TSG 101, CD 63, and CD9) in nSkM-Exos and sSkM-Exos via WB. *Data were shown as means ± SD*, *n* = 3. ∗*p* < 0.05; ∗∗*p* < 0.01; ∗∗∗*p* < 0.001; ∗∗∗∗*p* < 0.0001; ns, *no significant difference*

Cellular senescence is an inevitable physiological process. To investigate the effect of sSkM-Exos on NPCs, we established a stable senescent SkMCs model. CCK-8 assay was performed to screen optimal induction conditions: as the concentration of H₂O₂ increased, SkMCs viability exhibited a concentration-dependent decrease. After continuous treatment with 100 µM H₂O₂, cell viability remained at a low level with no significant recovery observed even as the treatment duration prolonged, indicating that this concentration could induce persistent cellular damage (Fig. 1B, C). Following H₂O₂-induced senescence in SkMCs, the proportion of SA-β-gal-positive cells was significantly increased (Fig. 1D, G). Additionally, the immunofluorescence intensity of the senescence-associated markers P16 and P21 was elevated (Fig. 1E)—a finding further corroborated by qPCR results (Fig. 1F). These experiments collectively confirmed the successful establishment of the senescent SkMCs model. SkM-Exos were successfully isolated via differential centrifugation (Fig. 1A). TEM images revealed that exosomes from both groups displayed the characteristic cup-shaped morphology. NTA showed that the particle sizes of normal skeletal muscle cell-derived exosomes (nSkM-Exos) and sSkM-Exos were 114.88 nm and 122.78 nm, respectively—consistent with the typical size range of exosomes (100–150 nm) (Fig. 1H). Protein concentrations of the isolated exosomes were quantified using a BCA assay (Fig. 1J). WB further verified the expression of exosome-specific markers (TSG101, CD9, and CD63) in both exosome preparations (Fig. 1K). To assess the uptake efficiency of exosomes by NPCs, NPCs were co-incubated with PHK26-labeled exosomes. Confocal microscopy observations showed that after 6 h and 12 h of co-incubation, the labeled exosomes were localized around and within NPCs, with their fluorescent signals showing a time-dependent dynamic pattern (Fig. 1I). Taken together, these results confirmed that sSkM-Exos were successfully isolated and could be efficiently internalized by NPCs.

### The regulatory effect of sSkM-Exos on the activity of NPCs

**Fig. 2:**
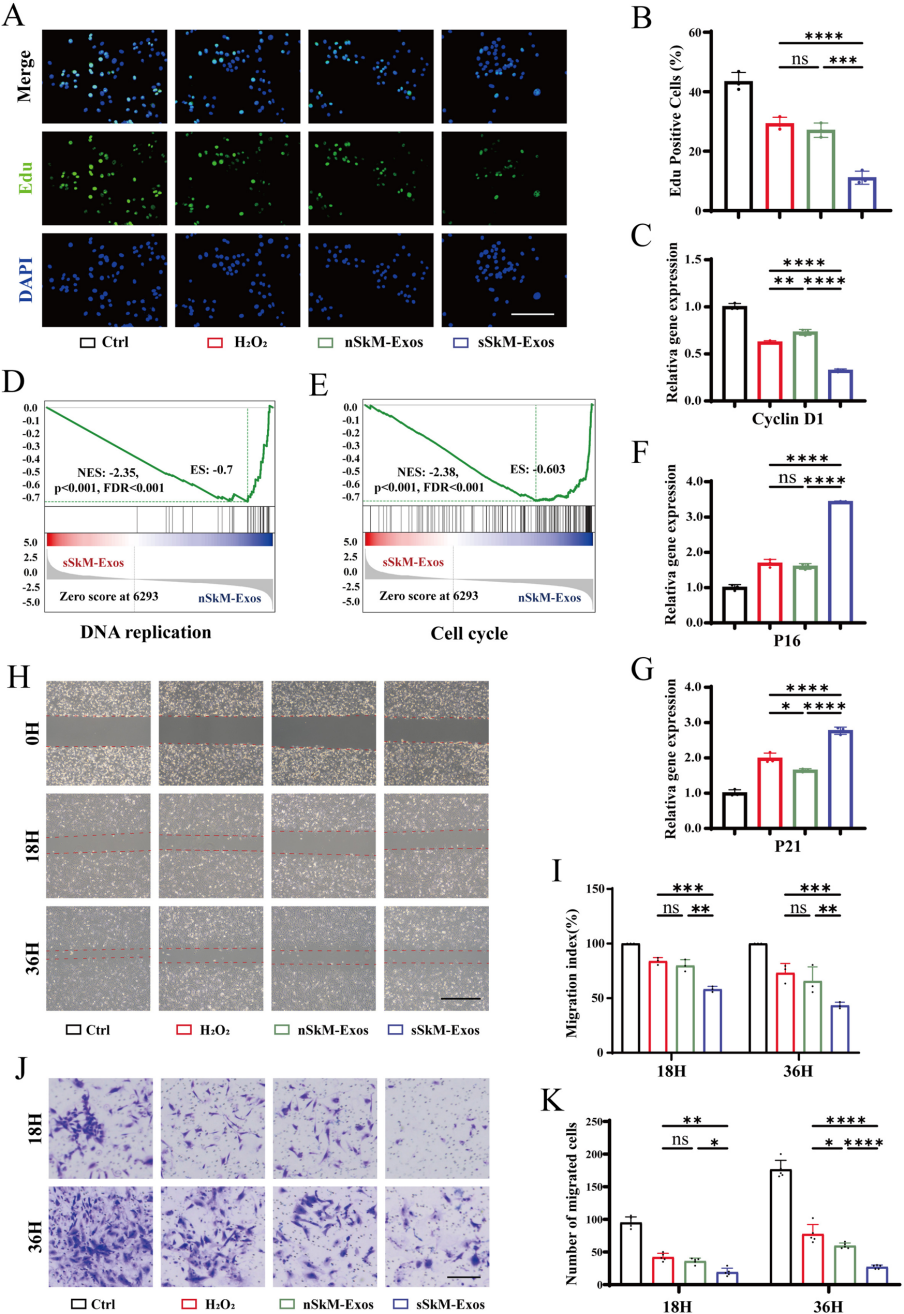
Regulatory effects of sSkM-Exos on NPCs activity. **A**, **B**. EdU staining images and quantitative analysis of NPCs treated with different interventions. Scale bar, 50 μm. **C**, **F**, **G**. Quantification of Cyclin D1, P16, and P21 protein expression. **D**, **E**. GSEA results of NPCs following treatment with sSkM-Exos and nSkM-Exos. **H**, **I**. Scratch wound healing assay images and quantitative analysis of NPCs treated with different interventions. Scale bar, 100 μm. **J**, **K**. Transwell migration assay images and quantitative analysis of NPCs treated with different interventions. Scale bar, 100 μm. *Data are presented as means ± SD*, *n* = 3. ∗*p* < 0.05; ∗∗*p* < 0.01; ∗∗∗*p* < 0.001; ∗∗∗∗*p* < 0.0001; ns, *no significant difference*

To systematically investigate the regulatory effects of sSkM-Exos on NPC viability, Fig. 2A and B showed that the EdU-positive rate was significantly decreased in the H₂O₂ group, indicating that oxidative stress significantly impairs the proliferative activity of NPCs. Notably, sSkM-Exos further reduced the EdU-positive rate, demonstrating that sSkM-Exos inhibit NPC proliferation. The results of qPCR revealed that after H₂O₂ treatment, the transcriptional level of Cyclin D1 (a core regulatory gene for proliferation) was significantly downregulated, while the transcriptional levels of senescence-associated marker genes P16 and P21 were significantly upregulated. Moreover, sSkM-Exos intervention further exacerbated these abnormal gene expression patterns, whereas nSkM-Exos exerted no significant reversing effect on the aforementioned results (Fig. 2C, F, G). Transcriptome sequencing revealed that “DNA replication” and “Cell cycle” signaling pathways were significantly enriched in the down-regulated gene set, which further confirmed that sSkM-Exos reduced the viability of NPCs (Fig. 2D, E). Wound healing assay and Transwell assay demonstrated that the H₂O₂ group exhibited impaired NPCs migration ability. Treatment with sSkM-Exos aggravated the inhibitory effect of H₂O₂ -induced migration in a time-dependent manner, while nSkM-Exos showed no effect on H₂O₂ -induced migration impairment (Fig. 2H-K). Collectively, these results indicate that sSkM-Exos exerts its regulatory effects by inhibiting proliferation-related pathways and disrupting the expression of proliferation-senescence genes.

### sSkM-Exos promote senescence and apoptosis of NPCs

**Fig. 3:**
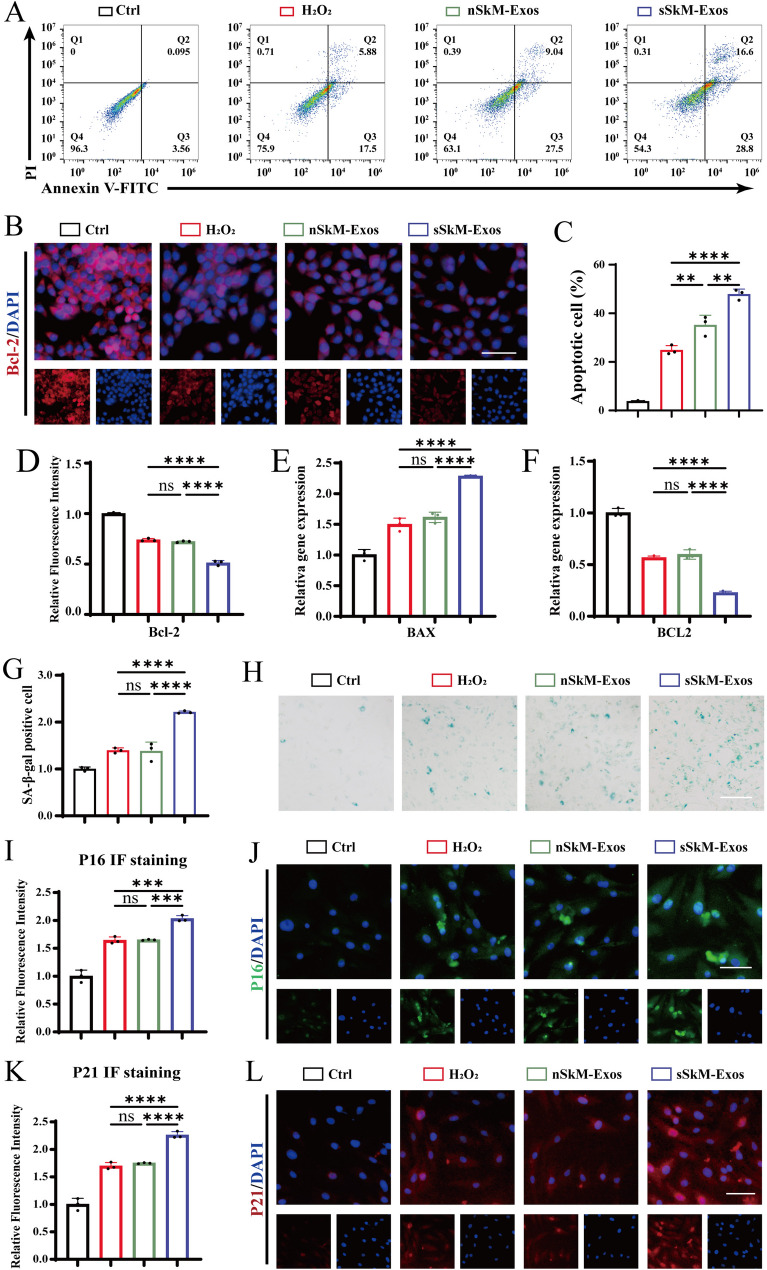
Effects of sSkM-Exos on senescence and apoptosis of nucleus pulposus cells. **A**, **C.** Apoptosis results and quantitative analysis of NPCs treated under different conditions. **B**, **D.** Representative fluorescence images and quantitative analysis of Bcl-2 in NPCs treated under different conditions. Scale bar, 50 μm. **E**, **F.** Quantification of BAX and BCL2 expression. **G**, **H.** SA-β-gal staining and quantitative analysis results of NPCs treated under different conditions. Scale bar, 100 μm. **I-L.** Representative fluorescence images and quantitative analysis of senescence markers in NPCs treated under different conditions. Scale bar, 50 μm. *Data are shown as means ± SD*, *n* = 3. ∗*p* < 0.05; ∗∗*p* < 0.01; ∗∗∗*p* < 0.001; ∗∗∗∗*p* < 0.0001; ns,* no significant difference*

To systematically assess the regulatory effects of sSkM-Exos on senescence and apoptosis of NPCs, relevant assay results demonstrated that compared with the H₂O₂ group, the apoptosis rate of NPCs in the sSkM-Exos group was further significantly elevated. This finding indicates that sSkM-Exos can exacerbate the oxidative stress-induced NPC apoptosis process (Fig. 3A, C). To clarify the molecular mechanism underlying apoptosis regulation, ICC staining for the anti-apoptotic protein Bcl-2 demonstrated that H₂O₂ treatment markedly decreased the fluorescence intensity of Bcl-2, and sSkM-Exos treatment further exacerbated the downregulation of this protein (Fig. 3B, D). This result was highly consistent with qPCR findings: in the H₂O₂ group, the expression of the pro-apoptotic gene BAX was upregulated while the anti-apoptotic gene BCL2 was downregulated, and sSkM-Exos treatment significantly exacerbated these aberrant gene expression profiles (Fig. 3E, F). For the assessment of cellular senescence phenotypes, SA-β-gal staining revealed that H₂O₂ treatment markedly increased the proportion of SA-β-gal-positive senescent cells, and sSkM-Exos treatment further significantly elevated this proportion (Fig. 3G, H). Immunocytochemistry (ICC) staining for the core senescence markers P16 and P21 further corroborated that H₂O₂ treatment markedly enhanced their fluorescence intensity, and sSkM-Exos treatment significantly exacerbated the upregulation of these two senescence markers (Fig. 3I-L). Collectively, these findings demonstrate that sSkM-Exos aggravate the senescence and apoptosis of NPCs.

### The effect of sSkM-Exos on the ECM metabolism of NPCs

**Fig. 4:**
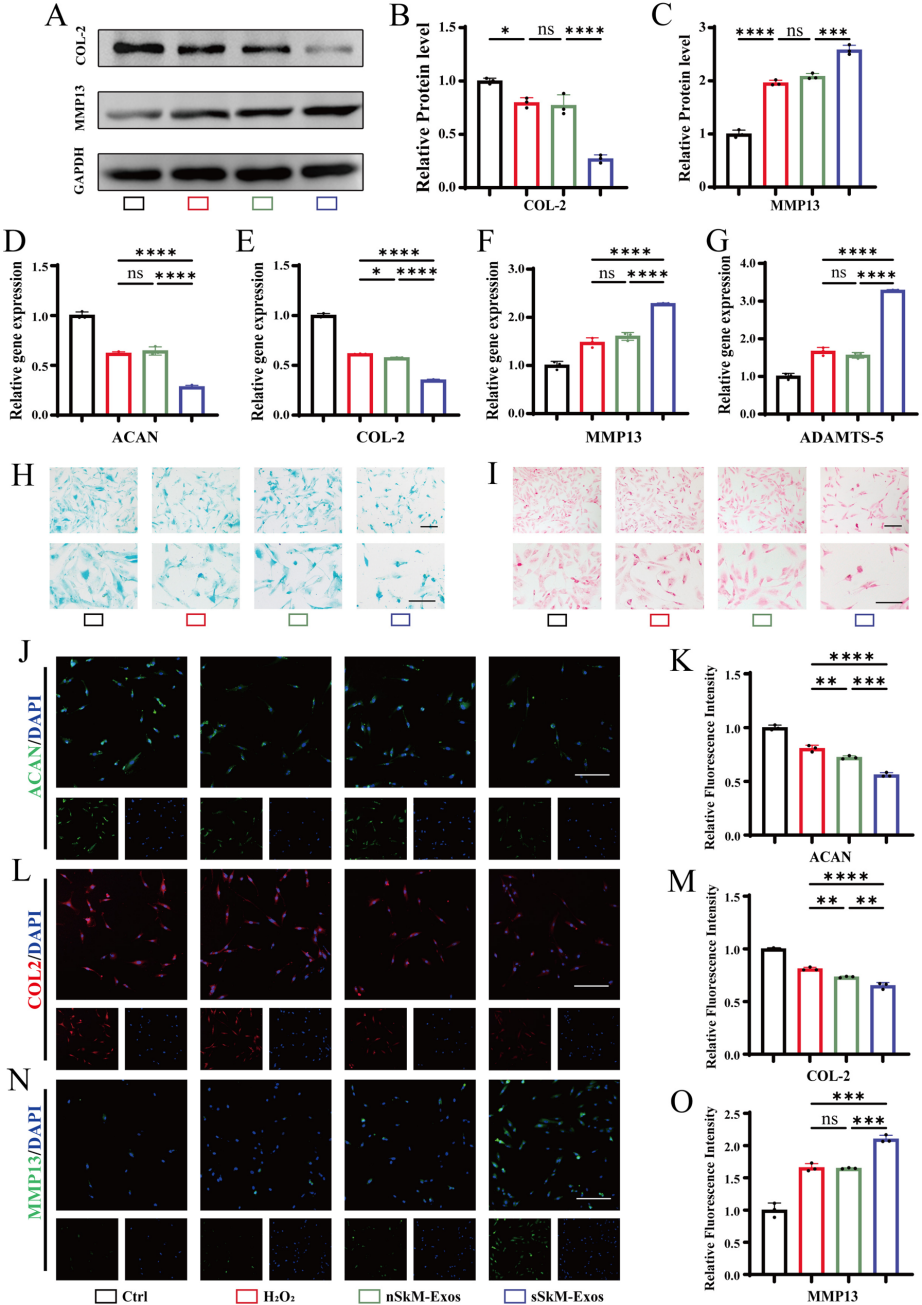
Effects of sSkM-Exos on ECM metabolism in NPCs. **A-C.** Expression of MMP13 and COL2 in NPCs was analyzed by WB. **D-G.** mRNA expression of ACAN, COL2, MMP13 and ADAMTS-5 was analyzed by RT-qPCR. **H**, **I.** Results of Alcian blue staining and safranin O staining of NPCs under different treatment conditions. **J-O.** Expression and quantitative analysis of ACAN, COL2 and MMP13 by immunofluorescence. Scale bar, 50 μm. *Data are shown as means ± SD*, *n* = 3. ∗*p* < 0.05; ∗∗*p* < 0.01; ∗∗∗*p* < 0.001; ∗∗∗∗*p* < 0.0001; ns,* no significant difference*

The above experiments confirmed that sSkM-Exos inhibit the proliferation of NPCs and aggravate their senescence and apoptosis. Next, we further investigated the role of sSkM-Exos in ECM metabolism during the progression of IVDD. At the protein level, WB analysis demonstrated that H₂O₂ treatment significantly downregulated type II collagen (COL2, a key ECM synthesis protein) and upregulated matrix metalloproteinase 13 (MMP13, a key ECM degradation enzyme); sSkM-Exos treatment further exacerbated this regulatory trend, suggesting that sSkM-Exos inhibit ECM synthesis while aggravating its degradation (Fig. 4A-C). The results of qPCR further corroborated these findings: in the H₂O₂ group, the transcriptional levels of ECM synthesis genes (ACAN, COL2) were decreased, while those of ECM degradation genes (MMP13, ADAMTS5) were increased; sSkM-Exos exacerbated this metabolic imbalance, whereas nSkM-Exos exerted negligible regulatory effects (Fig. 4D-G). Alcian blue and Safranin-O staining provided visual insights into the morphological changes of matrix synthesis in NPCs, offering key morphological evidence for the protein- and gene-level results. In the normal group, the cytoplasm and intercellular spaces of NPCs exhibited deep blue positive staining, characterized by extensive and uniform positive areas. In the H₂O₂ group, staining intensity was markedly attenuated and the positive area was diminished. In the sSkM-Exos group, staining intensity was further significantly lower than that in the H₂O₂ group, with only faint light-blue positive staining detected in local cytoplasmic regions. No significant difference in staining intensity was observed between the nSkM-Exos and H₂O₂ groups (Fig. 4H). Safranin-O staining showed that H₂O₂ reduced ECM secretion, and sSkM-Exos further inhibited this process (Fig. 4I). ICC staining results verified the intergroup differences observed in the aforementioned experiments (Fig. 4J-O). In conclusion, sSkM-Exos exerts a critical pro-degenerative role in the pathological progression of IVDD by perturbing the ECM metabolic homeostasis of NPCs.

### sSkM-Exos accelerate the pathological progression of IVDD

**Fig. 5:**
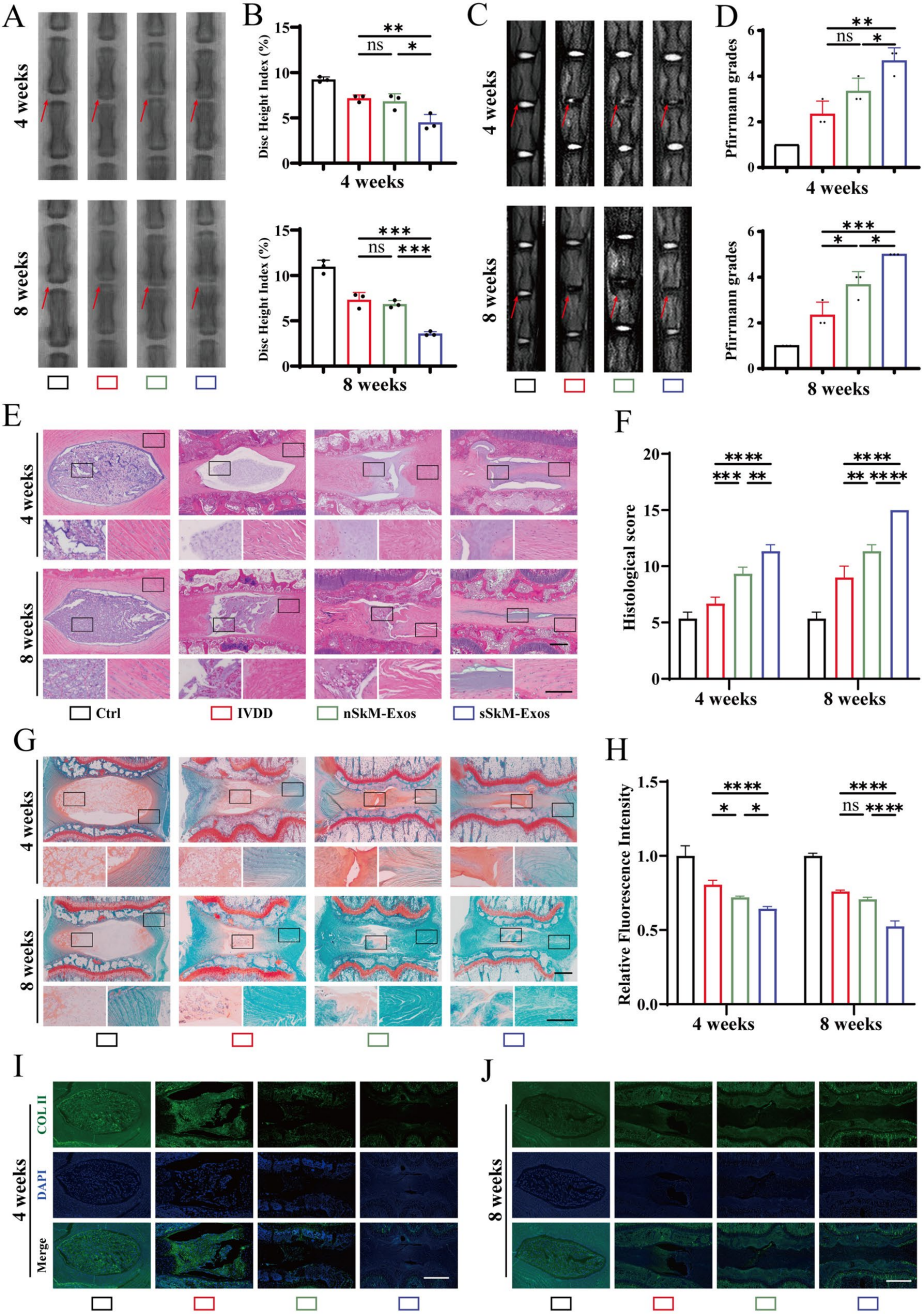
sSkM-Exos promote senescence of NPCs *in vivo.* **A**, **B**. X-ray imaging and quantitative analysis of rat tails at 4 and 8 weeks post-surgery. **C**, **D**. T2-weighted MRI scans and quantitative analysis of rat tails at 4 and 8 weeks post-surgery. **E-G**. HE and Safranin O staining of rat tail intervertebral discs and histological scoring results. Scale bar, 1000 μm. **H-J**. Representative immunofluorescent images of IVDD-specific markers following sSkM-Exos intervention. Scale bar, 50 μm. *Data are presented as means ± SD*, *n* = 3. ∗*p* < 0.05; ∗∗*p* < 0.01; ∗∗∗*p* < 0.001; ∗∗∗∗*p* < 0.0001; ns,* no significant difference*

*In vivo*, we established a rat IVDD model to systematically evaluate the impact of sSkM-Exos on pathological progression. Imaging assessments revealed that sSkM-Exos intervention markedly exacerbated IVDD progression: specifically, X-ray images showed aggravated intervertebral disc space narrowing, while T2-weighted MRI scans exhibited significantly reduced signal intensity, as shown in Table S3 (Supporting Information). Notably, this degenerative phenotype was further exacerbated as the observation period extended from 4 to 8 weeks (Fig. 5A-D). HE staining of intervertebral disc sections demonstrated prominent morphological changes in the IVDD group, as shown in Table S4 (Supporting Information). At 4 weeks, structural disorganization, decreased nucleus pulposus cell (NPC) density, and blurred annulus fibrosus-nucleus pulposus boundary were observed; these degenerative features were further aggravated at 8 weeks. Importantly, sSkM-Exos intervention further exacerbated this degenerative trend (Fig. 5E, F). Safranin O-fast green staining visually reflected ECM content in intervertebral discs: in the IVDD group, Safranin O staining intensity in the nucleus pulposus region was attenuated, indicating ECM loss. After sSkM-Exos injection, staining intensity was further diminished, and the extent of matrix depletion at 8 weeks was more severe than that at 4 weeks (Fig. 5G). Paraffin-section immunofluorescence staining for COL2 (a key ECM component, Fig. 5H-J) further confirmed ECM synthetic capacity: COL2 positive expression in the IVDD group gradually decreased over time. sSkM-Exos administration significantly suppressed COL2 expression in intervertebral disc tissues at both 4 and 8 weeks, whereas COL2 expression in the nSkM-Exos group showed no significant difference compared with the IVDD group. Collectively, these *in vivo* findings confirm that sSkM-Exos significantly promote IVDD progression by exacerbating disc height loss, structural disruption, and ECM degradation.

### sSkM-Exos promote the senescence of NPCs through the MAPK signaling pathway

**Fig. 6:**
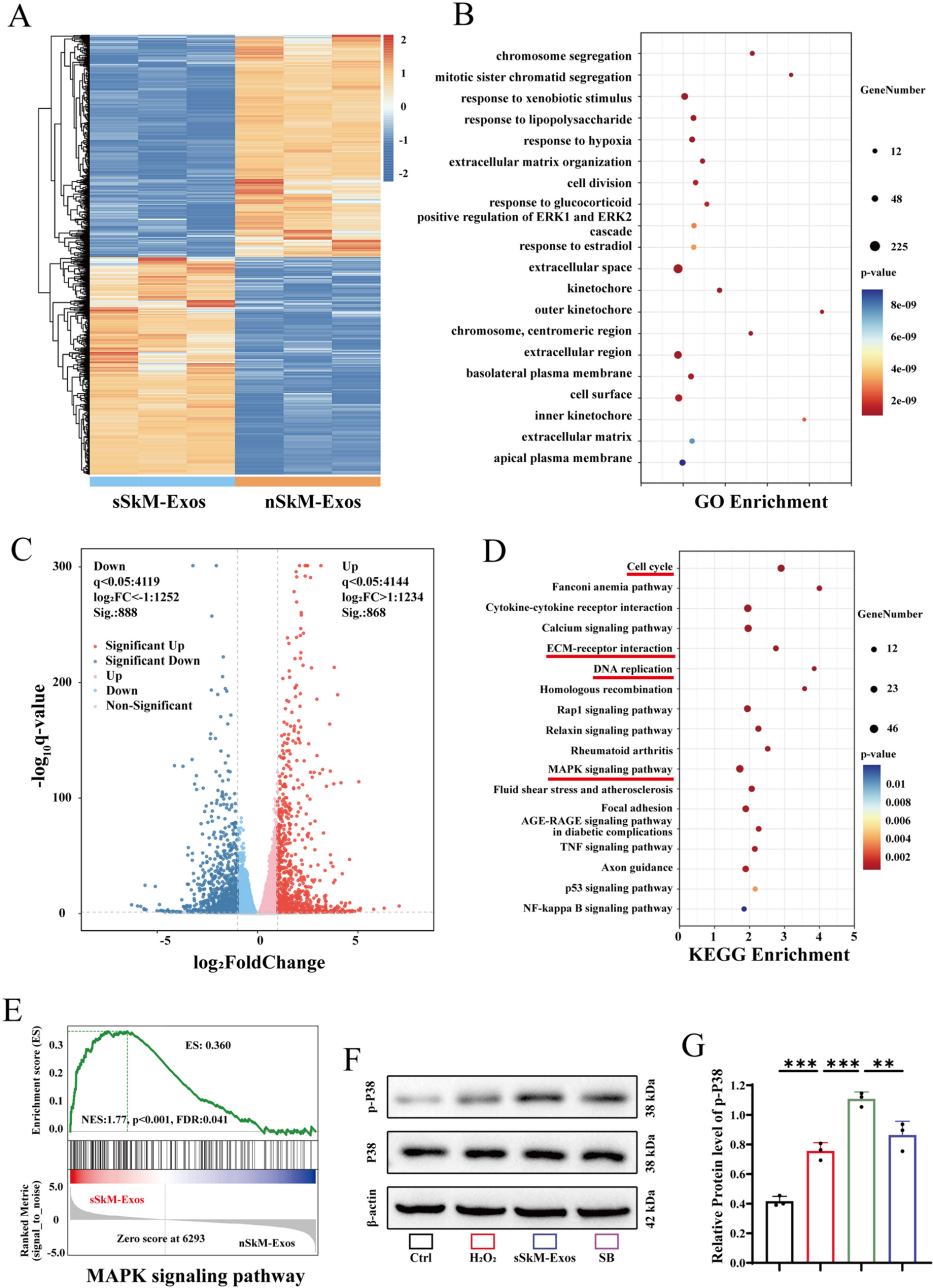
sSkM-Exos promote the senescence of NPCs through the p38MAPK signaling pathway. **A**, **C.** Volcano plots and heat maps of all DEGs (|log_2_FC| > 1) after sSkM-Exos and nSkM-Exos intervention in NPCs. **B**, **D.** Results of KEGG enrichment analysis and GO enrichment analysis of DEGs. **E.** GSEA results of DEGs. **F**, **G.** WB analysis shows that sSkM-Exos activate the p38MAPK signaling pathway. *Data are shown as means ± SD*, *n* = 3. ∗*p* < 0.05; ∗∗*p* < 0.01; ∗∗∗*p* < 0.001; ∗∗∗∗*p* < 0.0001; ns, *no significant difference*

To explore the molecular mechanism by which sSkM-Exos regulate IVDD, two groups of exosomes were separately added to H₂O₂-pretreated nucleus pulposus cells (NPCs). After 24 h of incubation, RNA sequencing (RNA-Seq) was performed to systematically analyze the differential characteristics of transcriptomic expression profiles. Differential gene screening revealed distinct expression clustering between the sSkM-Exos and nSkM-Exos groups, indicating that sSkM-Exos exert specific transcriptomic regulatory effects on NPCs. A total of 1,756 significantly DEGs were identified in the sSkM-Exos-treated group, including 868 upregulated and 888 downregulated genes, with expression levels significantly distinct from those in the nSkM-Exos group (Fig. 6A, C). For functional enrichment analysis, Gene Ontology (GO) enrichment results demonstrated that DEGs modulated by sSkM-Exos were predominantly enriched in biological processes such as “regulation of cell division”, “extracellular matrix (ECM) assembly and degradation”, and “oxidative stress response”. They were also enriched in cellular components (e.g., “ECM component”) and molecular functions (e.g., “protein kinase activity”) (Fig. 6B). Kyoto Encyclopedia of Genes and Genomes (KEGG) pathway enrichment analysis further illustrated that DEGs were significantly enriched in pathways closely associated with cell proliferation and ECM metabolism, including “Cell Cycle Regulation”, “ECM-Receptor Interaction”, and “Mitogen-Activated Protein Kinase (MAPK) Signaling Pathway” (Fig. 6D). To pinpoint the core regulatory pathway, Gene Set Enrichment Analysis (GSEA) showed that MAPK signaling pathway-related gene sets in the sSkM-Exos-treated group were significantly enriched and activated (NES = 1.77, *P* < 0.001, FDR q-value < 0.01), indicating that this pathway likely serves as a key mediator for sSkM-Exos to regulate NPC function (Fig. 6E). Prior studies have established that P38, a key component of the MAPK signaling pathway, exerts a pivotal regulatory role in IVDD progression. To validate the reliability of RNA-Seq results, WB analysis was performed to measure the phosphorylation status of P38 (a core MAPK pathway molecule). The protein expression of phosphorylated P38 (p-P38) in sSkM-Exos-treated NPCs was significantly higher than that in both the control and nSkM-Exos-treated groups, while total P38 expression remained unchanged. This finding directly validated the activating effect of sSkM-Exos on the MAPK signaling pathway at the post-translational modification level (Fig. 6F, G).

### Inhibition of the p38MAPK signaling pathway blocks the sSkM-Exos-mediated pro-senescence effect on NPCs

**Fig. 7:**
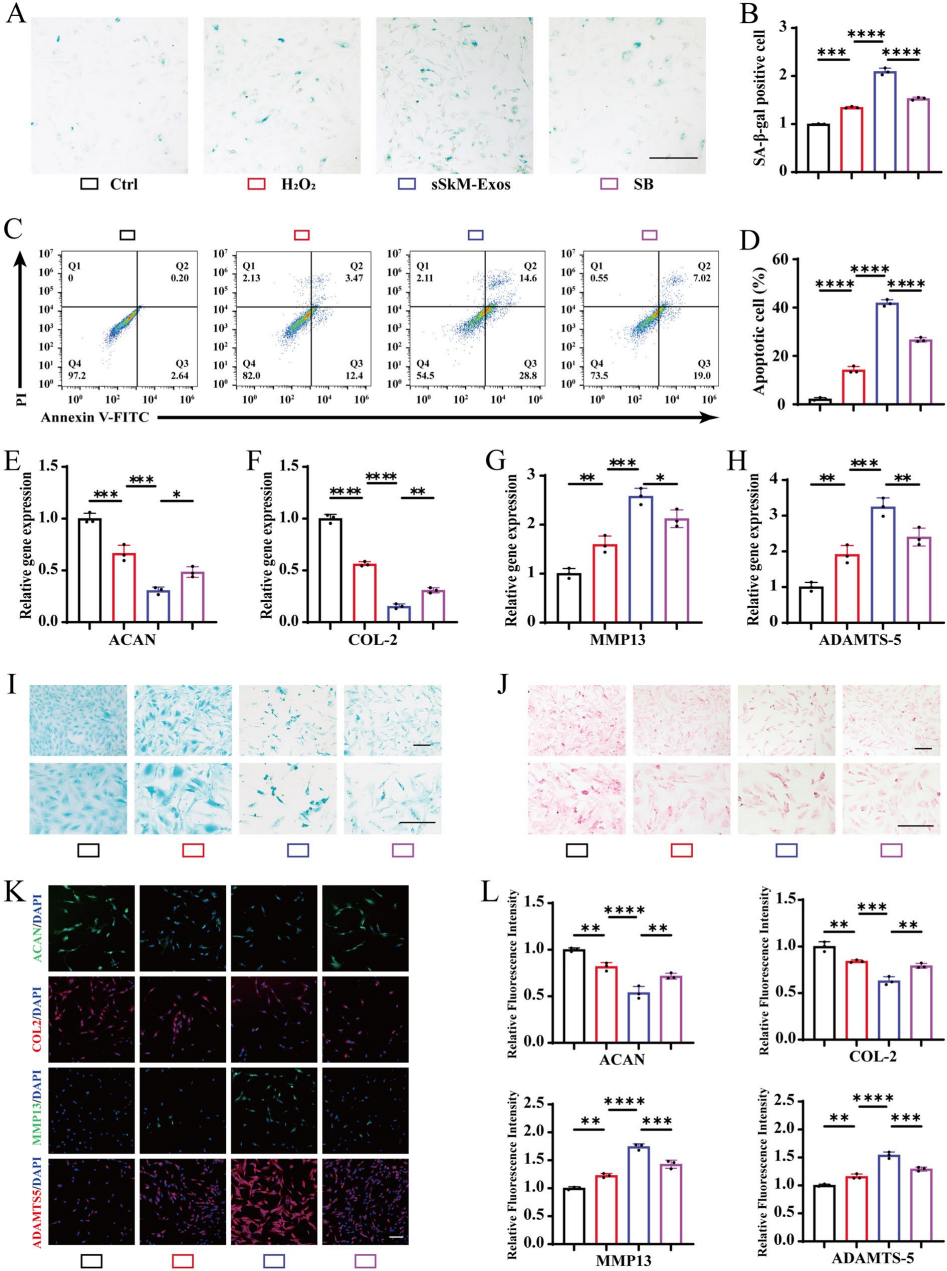
Inhibition of the p38MAPK signaling pathway blocks the pro-aging effect of sSkM-Exos on NPCs. **A**, **B.** Results of SA-β-gal staining and quantitative analysis of NPCs under different treatment conditions. Scale bar, 100 μm. **C**, **D.** Apoptosis results and quantitative analysis of NPCs under different treatment conditions. **E-H.** mRNA expression of ACAN, COL2, MMP13 and ADAMTS-5 analyzed by RT-qPCR. **I**, **J.** Results of Alcian blue staining and safranin O staining of NPCs under different treatment conditions. **K**, **L.** Expression of ACAN, COL2, MMP13 and ADAMTS-5 detected by immunofluorescence and quantitative analysis. Scale bar, 50 μm. *Data are presented as means ± SD*, *n* = 3. ∗*p* < 0.05; ∗∗*p* < 0.01; ∗∗∗*p* < 0.001; ∗∗∗∗*p* < 0.0001; ns, *no significant difference*

To elucidate the role of the p38MAPK signaling pathway in sSkM-Exos-mediated pro-senescence of NPCs, we treated NPCs with sSkM-Exos either alone or in combination with the p38-specific inhibitor SB203580 (SB). According to prior research, SB was initially dissolved in dimethyl sulfoxide (DMSO) before being diluted with culture medium, ensuring the final concentration of DMSO remained below 0.1% [30]. For cellular senescence assessment, SA-β-gal staining revealed that co-treatment with SB significantly abrogated sSkM-Exos-mediated pro-senescence, confirming that sSkM-Exos exacerbate NPC senescence via p38MAPK pathway activation (Fig. 7A, B). Consistent with this, sSkM-Exos treatment significantly increased NPC apoptosis, which was reversed by SB intervention (Fig. 7C, D). In the transcriptional level detection of ECM-related genes, the qPCR analysis results showed that H₂O₂ treatment significantly downregulated the mRNA expression of COL2 and upregulated the expression of MMP13. The sSkM-Exos treatment further exacerbated this imbalance between synthesis and degradation, while the SB intervention effectively restored the transcriptional level of COL2 and significantly inhibited the high expression of MMP13 (Figs. 7E-H). At the morphological level, Alcian blue and Safranin O staining visually confirmed that sSkM-Exos suppressed matrix synthesis, whereas SB intervention rescued ECM secretion capacity (Fig. 7I, J). ICC staining for marker localization further verified that H₂O₂ significantly reduced the fluorescence signals of ACAN and COL2, while enhancing those of MMP13 and ADAMTS5, sSkM-Exos exacerbated this phenotypic imbalance, whereas SB enhanced the signals of synthetic markers and suppressed the expression of degradative markers (Fig. 7K, L). Collectively, these findings demonstrate that sSkM-Exos exacerbate H₂O₂-induced NPC senescence, apoptosis, and ECM metabolic imbalance via p38MAPK pathway activation. Importantly, the p38MAPK inhibitor SB can reverse sSkM-Exos-mediated NPC functional impairment by specifically targeting this pathway, providing experimental evidence for the mechanism investigation and therapeutic intervention of IVDD.

### Inhibition of the p38MAPK signaling pathway delays the pathological progression of IVDD

**Fig. 8:**
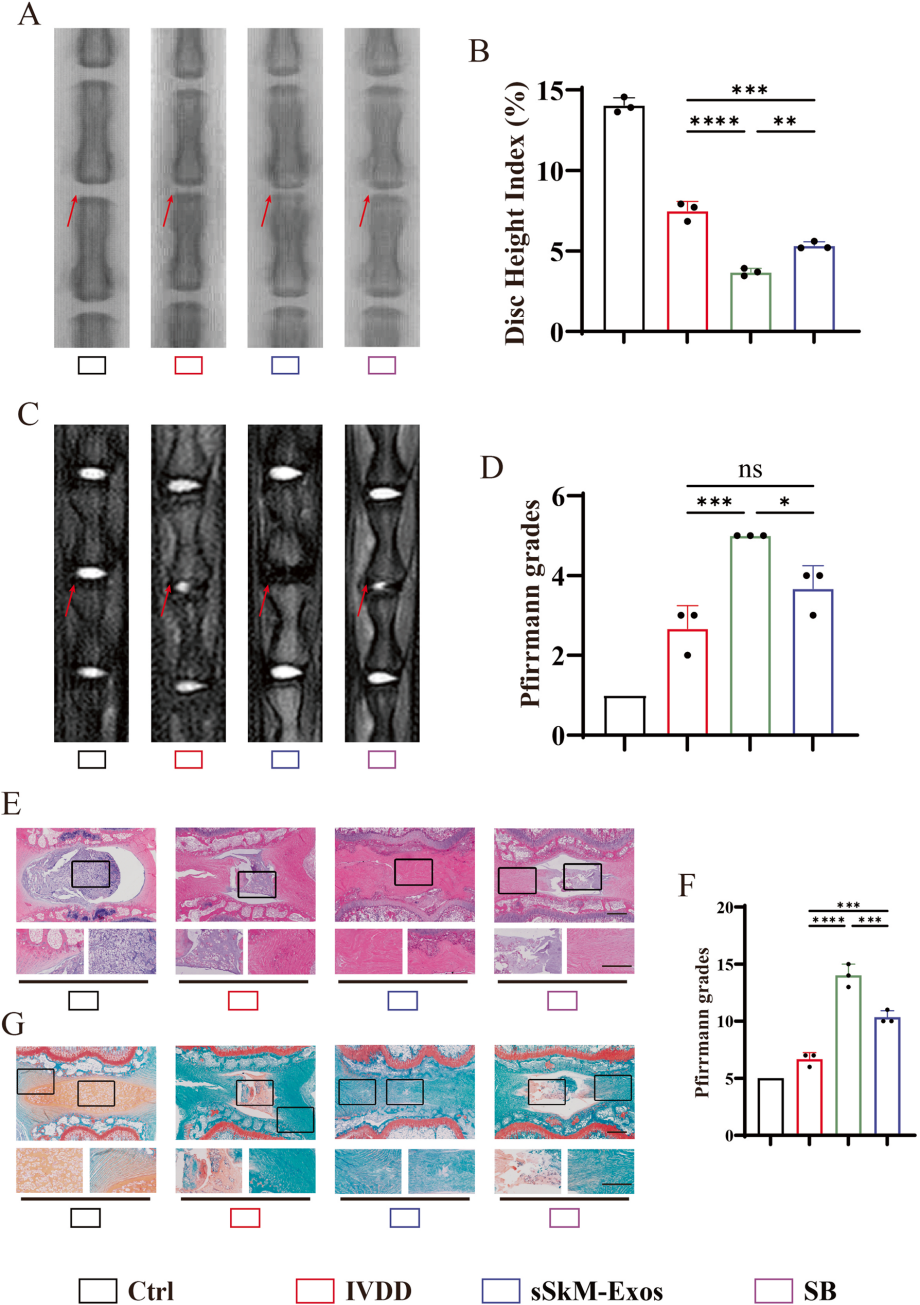
After inhibition of the p38MAPK signaling pathway, the pathological progression of IVDD mediated by sSkM-Exos was delayed. **A-D.** X-ray and MRI examinations and quantitative analyses of the rat tails. **E-G.** HE and safranin O staining and histological grading of the intervertebral discs in the rat tails. Scale bar, 1000 μm. *Data are shown as means ± SD*, *n* = 3. ∗*p* < 0.05; ∗∗*p* < 0.01; ∗∗∗*p* < 0.001; ∗∗∗∗*p* < 0.0001; ns,* no significant difference*

*In vivo* experiments further verified that inhibition of the p38MAPK signaling pathway can block the sSkM-Exos-mediated pathological progression of IVDD. In imaging assessments, X-ray images clearly showed that the SB group significantly reversed sSkM-Exos-induced intervertebral disc height reduction (Fig. 8A, B). The intergroup differences observed in T2-weighted MRI images were consistent with the X-ray findings (Fig. 8C, D). Furthermore, HE and Safranin O/Fast Green staining results showed that compared with the IVDD group, intervertebral disc structural damage was further aggravated in the sSkM-Exos-treated group. Notably, compared with the sSkM-Exos group, the SB group exhibited significantly improved disc structural organization, a marked increase in NPCs density, and a significant reduction in degeneration score (Fig. 8E-G). Collectively, these findings demonstrate that sSkM-Exos significantly exacerbate H₂O₂-induced caudal IVDD in rats by activating the p38MAPK pathway. In contrast, the p38MAPK inhibitor SB can effectively reverse sSkM-Exos-mediated degenerative processes through targeted inhibition of this pathway, providing experimental evidence for *in vivo* IVDD intervention.

## Discussion

IVDD is a primary etiological factor of chronic LBP, severely compromising the quality of life of hundreds of millions of individuals globally [31, 32]. For decades, research into the pathological mechanisms of IVDD has primarily focused on the mechanical regulatory axis. From a traditional biomechanical perspective, the chronic accumulation of mechanical loading is considered the central driver of structural decay within the disc. This process involves more than just fluctuations in internal pressure; it is often accompanied by physical damage to the annulus fibrosus, such as micro-cracks or tears [33, 34]. These structural failures subsequently lead to the physical disruption of the blood-disc barrier, ultimately triggering the senescence and dysfunction of NPCs and compromising both spinal stability and motor function [35].

The innovation of this study lies in proposing a potential cross-tissue regulatory axis: muscle aging-exosomes-IVDD. While previous research primarily emphasized mechanical loading, our data suggest that sSkM-Exos carry pro-senescence signals that may contribute to the modulation of the NPC phenotype. This mechanism provides a supplementary perspective to the multifactorial pathogenesis of IVDD, shifting the focus toward systemic microenvironmental influences. Existing evidence indicates that sSkM-Exos carry specific pro-senescence factors and pro-inflammatory signaling molecules [36, 37]. As a potential myogenic messenger of senescence information, the pathological signals enriched in sSkM-Exos may activate inflammatory signaling pathways once they enter the intervertebral disc. This inflammatory cascade could then serve as a contributor to the phenotypic transition of NPCs, potentially triggering their programmed senescence. Collectively, these pathological events may establish a senescence-driven and inflammation-amplified cycle within the disc, potentially accelerating the progression of IVDD. This cross-tissue and trans-cellular signaling mechanism suggests a connection between muscle aging and disc degeneration, providing a critical cross-tissue regulatory component to the multifactorial pathogenesis of IVDD.

This study demonstrates that sSkM-Exos contribute to IVDD progression via three-pronged pathological effects: proliferation inhibition, senescence induction, and metabolic disruption. In vitro experiments indicated that sSkM-Exos affected the proliferative activity and senescent status of NPCs, exerting a synergistic effect with oxidative stress-induced damage. This suggests that as a carrier of the SASP, sSkM-Exos may exacerbate NPC functional deterioration through “intercellular senescence transmission.” Regarding ECM metabolism, sSkM-Exos disrupted the balance between synthesis and degradation. *In vivo* experiments further validated that sSkM-Exos intervention promoted degenerative changes in rat caudal intervertebral discs. Transcriptome sequencing and functional enrichment analysis pointed toward the MAPK signaling pathway, specifically the phosphorylation of p38MAPK. Subsequent intervention experiments confirmed that blocking the p38MAPK pathway significantly mitigated sSkM-Exos-induced NPC dysfunction. This identifies the p38MAPK pathway as a key regulatory mediator—though likely not the only one—of these pro-degenerative effects.

The composition and function of skeletal muscle-derived exosomes are not static but dynamically regulated by the muscle’s intrinsic metabolic state and external stimuli. Previous studies have demonstrated that exercise induces skeletal muscle cells to secrete exosomes enriched with specific miRNAs (e.g., miR-1, miR-133a, and miR-206) and anti-inflammatory proteins (e.g., IL-10 and HSP70) [38–41]. These components are delivered to immune cells (e.g., macrophages) via paracrine or endocrine pathways, promoting their polarization toward the anti-inflammatory (M2) phenotype, thereby suppressing the secretion of pro-inflammatory factors (e.g., TNF-α and IL-6) and mitigating local and systemic inflammation [42]. During aging, similar metabolic shifts in skeletal muscle (e.g., a glycolytic shift) may result in the enrichment of pro-inflammatory and pro-senescent molecular cargo in its exosomes [43, 44]. The senescent skeletal muscle microenvironment modulates the biological activity of the exosomes it secretes, transforming them from potential homeostasis-maintaining signaling carriers into pro-degenerative effector molecules. Compared with exosomes derived from young muscle cells, those from senescent muscle cells significantly upregulate the expression of senescence markers (e.g., p53 and p21) in nucleus pulposus cells [36]. This phenomenon suggests that senescent muscle tissue can “infect” adjacent tissues with senescence via exosomes. The universal principle that “pathological muscles derive pathological vesicles” is corroborated in this mechanism, and its potential applicability may extend to the pathological context of IVDD.

The “sSkM-Exos/p38MAPK” regulatory axis identified in this study provides critical molecular evidence for elucidating the synergistic progression mechanism between PM aging and IVDD. Epidemiological data demonstrate that skeletal muscle aging (sarcopenia) and IVDD exhibit a significant age-dependent comorbidity pattern, yet the pathological link between these two conditions has long remained elusive [45]. The traditional perspective posits that PM atrophy promotes IVDD by altering the spinal mechanical environment (e.g., increasing intradiscal pressure)—a mechanism termed the “mechanical regulatory axis” [46]. In contrast, this study confirms that sSkM-Exos are internalized by NPCs and induce NPCs “non-autonomous senescence” via activating the p38MAPK pathway, thereby establishing a cross-tissue pathological regulatory axis: “muscle aging - exosome transmission - IVDD”. This finding clarifies that senescence-related humoral signals may exert an independent and pivotal regulatory role in IVDD progression.

Despite these insights, several limitations remain. First, the specific molecular cargo within sSkM-Exos mediating p38MAPK activation has yet to be identified. Second, the H₂O₂-induced SkMC senescence model may not fully capture the chronic secretory profile of natural aging. Third, the exosome concentrations used and the rat caudal disc model may not perfectly replicate physiological levels or human lumbar biomechanics. Future studies using human samples and long-term aging models are essential to validate these findings’ clinical relevance.

## Conclusion

In conclusion, our findings suggest that sSkM-Exos can be internalized by NPCs, contributing to NPC senescence and ECM imbalance, likely through the activation of the p38MAPK signaling pathway. These results provide a molecular perspective that supplements the traditional biomechanical model of IVDD pathogenesis under experimental conditions. While further validation in human clinical samples is required, this study highlights the sSkM-Exos/p38MAPK axis as a potential target for the development of future IVDD intervention strategies.

## Supplementary Information

Supplementary Material 1

## Data Availability

Research data are available from the corresponding author upon reasonable request.
