# Supplementary material for: Exosomes derived from senescent skeletal muscle cells aggravate nucleus pulposus cell metabolic dysregulation via p38MAPK pathway for promoting intervertebral disc degeneration
Source: BMC Musculoskelet Disord. 2026 Apr 16;27:512. doi: 10.1186/s12891-026-09816-8 (PMC13261835; doi:10.1186/s12891-026-09816-8)

**Exosomes derived from senescent skeletal muscle cells aggravate nucleus pulposus cell metabolic dysregulation via p38MAPK pathway for promoting intervertebral disc degeneration**

Xiaowei Ma^a, b, c,1^, Weiqi Zhang^a, b, c,1^, Han Yin^d^, Dazhuang Miao^a, b, c^, Xianda Gao^a^, Chunxu Fu^e^, Wei Chen^a^, Zhiyong Hou^a^, Qi Zhang^a^, Yingze Zhang^a, b, c, f,^ **, Di Zhang^a,^ *

^a^ Department of Orthopaedic Surgery, Hebei Medical University Third Hospital, Shijiazhuang, Hebei Province, 050051, China.

^b^ NHC Key Laboratory of Intelligent Orthopaedic Equipment, Hebei Medical University Third Hospital, Shijiazhuang, Hebei Province, 050051, China.

^c^ Orthopaedic Research Institution of Hebei Province, Hebei Medical University Third Hospital, Shijiazhuang, Hebei Province, 050051, China.

^d^ Department of Orthopaedic, Union Hospital, Tongji Medical College, Huazhong University of Science and Technology, Wuhan, Hubei Province, 430022, China.

^e^ School of Medicine, Nankai University, Tianjin 300071, China.

^f^ Chinese Academy of Engineering, Beijing, 100088, China

^*^ Corresponding author.

^**^ Corresponding author.

E-mail addresses: dryingzezhang@163.com (Y. Zhang), 38300320@hebmu.edu.cn (D. Zhang).

^1^ Xiaowei Ma and Weiqi Zhang are co-first authors who equally contributed to this manuscript.

**Supplementary Data**

**Supplementary Tables**

**Table S1.** Primer sequence used in the PCR experiment

| RNA sequence | |
| --- | --- |
| ACAN-Forward | TGGCCTGCCTGACTTTAGTG |
| ACAN-Reverse | CCTGAACCACTGACGCTGAT |
| COL2-Forward | GCAGCAAGAGCAAGGAGAAGAA |
| COL2-Reverse | CAGTGGACAGTAGACGGAGGAA |
| MMP13- Forward | CAAGCAGCTCCAAAGGCTAC |
| MMP13- Reverse | TGGCTTTTGCCAGTGTAGGT |
| GAPDH- Forward | GGTGAAGGTCGGTGTGAACG |
| GAPDH- Reverse | CTCGCTCCTGGAAGATGGTG |
| P16- Forward | GACCTAAGCGTACCGTCCAGAG |
| P16- Reverse | GAGAGCAGCAGATCACCAGATTAAC |
| P21- Forward | TGGGGAGAGGAGCTGGTGTTGT |
| P21- Reverse | CCGCCCCCTCCTCTAGCTGT |
| ADAMTS5- Forward | GCCCTTCTTCCTGCTGTTC |
| ADAMTS5- Reverse | GCTGTAGTCCTTGGTGGTCA |
| Cyclin-D1-Forward | CACGGCTCACGCTTACCTCA |
| Cyclin-D1-Reverse | ACTTGCGCGTCACAGGACAG |
| BAX-Forward | GCTTCAGGGTTTCATCCAGG |
| BAX-Reverse | CAGTTGAAGTTGCCGTCAGA |
| BCL2-Forward | GGTGGGGTCATGTGTGTGG |
| BCL2-Reverse | CGGTTCAGGTACTCAGTCATCC |

**Table S2.** Chemicals and Antibodies

| **Name** | **Dilution Ratio** | **Application** | **Source** |
| --- | --- | --- | --- |
| GAPDH | 1:10,000 | WB | Proteintech |
| ACAN | 1:1,000 | WB | Proteintech |
| COL-2 | 1:1,000 | WB | Proteintech |
| ADAMTS-5 | 1:1,000 | WB | Beyotime |
| MMP13 | 1:1000 | WB | Beyotime |
| CD9 | 1:1,000 | WB | Proteintech |
| CD63 | 1:1,000 | WB | Proteintech |
| TSG101 | 1:1,000 | WB | Proteintech |
| P38 | 1:1,000 | WB | Beyotime |
| p-P38 | 1:1,000 | WB | Beyotime |
| ACAN | 1:200 | ICC | Proteintech |
| COL-2 | 1:200 | ICC | Proteintech |
| ADAMTS-5 | 1:200 | ICC | Beyotime |
| MMP13 | 1:200 | ICC | Beyotime |

**Table S3.** Pfirrmann classification

| Grade | T2 Signal Intensity | Nucleus Pulposus-Annulus Fibrosus Boundary | Intervertebral Disc Height | Structural Uniformity |
| --- | --- | --- | --- | --- |
| Ⅰ | High signal (similar to cerebrospinal fluid) | Clearly distinguishable | Normal | Homogeneous |
| Ⅱ | Slightly high signal(higher than muscle) | Clear | Normal | Inhomogeneous with or  without horizontal bands |
| Ⅲ | Moderate signal (similar  to muscle) | Partially blurred | Normal or slightly decreased | Inhomogeneous, gray |
| Ⅳ | Low signal | Completely blurred | Moderately decreased | Inhomogeneous, gray to black |
| Ⅴ | Extremely low signal(similar to cortical bone) | Indistinguishable | Significantly collapsed | Inhomogeneous, gray toblack |

**Table S4.** Histological Grading Scale

| Category | Grade Description |
| --- | --- |
| Cellularity of the anulus fibrosus | Fibroblasts comprise > 75% of cells |
|  | Neither fibroblasts nor chondrocytes comprise > 75% of cells |
|  | Chondrocytes comprise > 75% of cells |
| Morphology of the anulus fibrosus | Well-organized collagen lamellae (no ruptured/serpentine fibers) |
|  | Inward bulging/ruptured/serpentine fibers in < 1/3 of the anulus |
|  | Inward bulging/ruptured/serpentine fibers in > 1/3 of the anulus |
| Border (anulus fibrosis nucleus pulposus) | Normal (no interruption) |
|  | Minimal interruption |
|  | Moderate/severe interruption |
| Cellularity of the nucleus pulposus | Normal: Stellar-shaped cells evenly distributed |
|  | Slight cell count decrease + mild clustering |
|  | Moderate/severe cell count decrease (> 50%)+ clustered cells separated by dense proteoglycans |
| Morphology of the nucleus pulposus | Round (> 1/2 of disc arca in midsagittal sections) |
|  | Rounded/irregular (1/4-1/2 of disc area in midsagittal sections) |
|  | lrregular (< 1/4 of disc area in midsagittal sections) |

**Supplementary Figures**

**Figure S1.** Measurement of Disc Height Index (DHI) for Intervertebral Space Height in X-ray Images. DHI=2×(D+E+F)/(A+B+C+G+H+I)


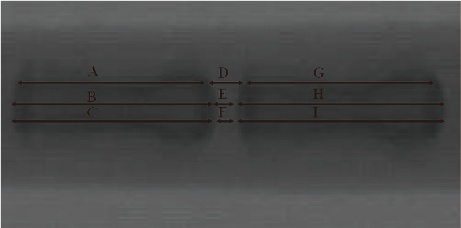


Supplementary Material: Original Blots

Fig. 4A:


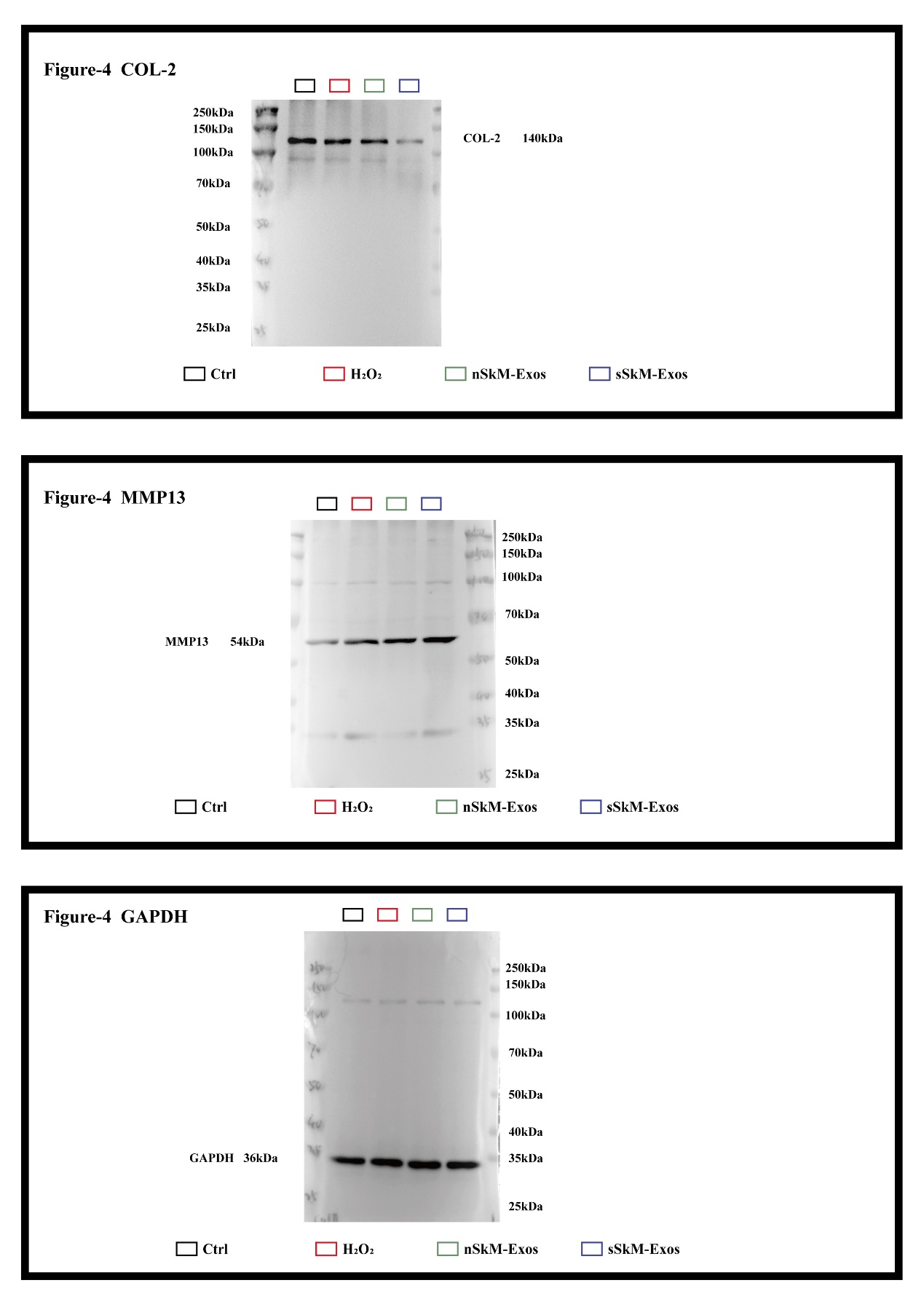


Fig. 6F


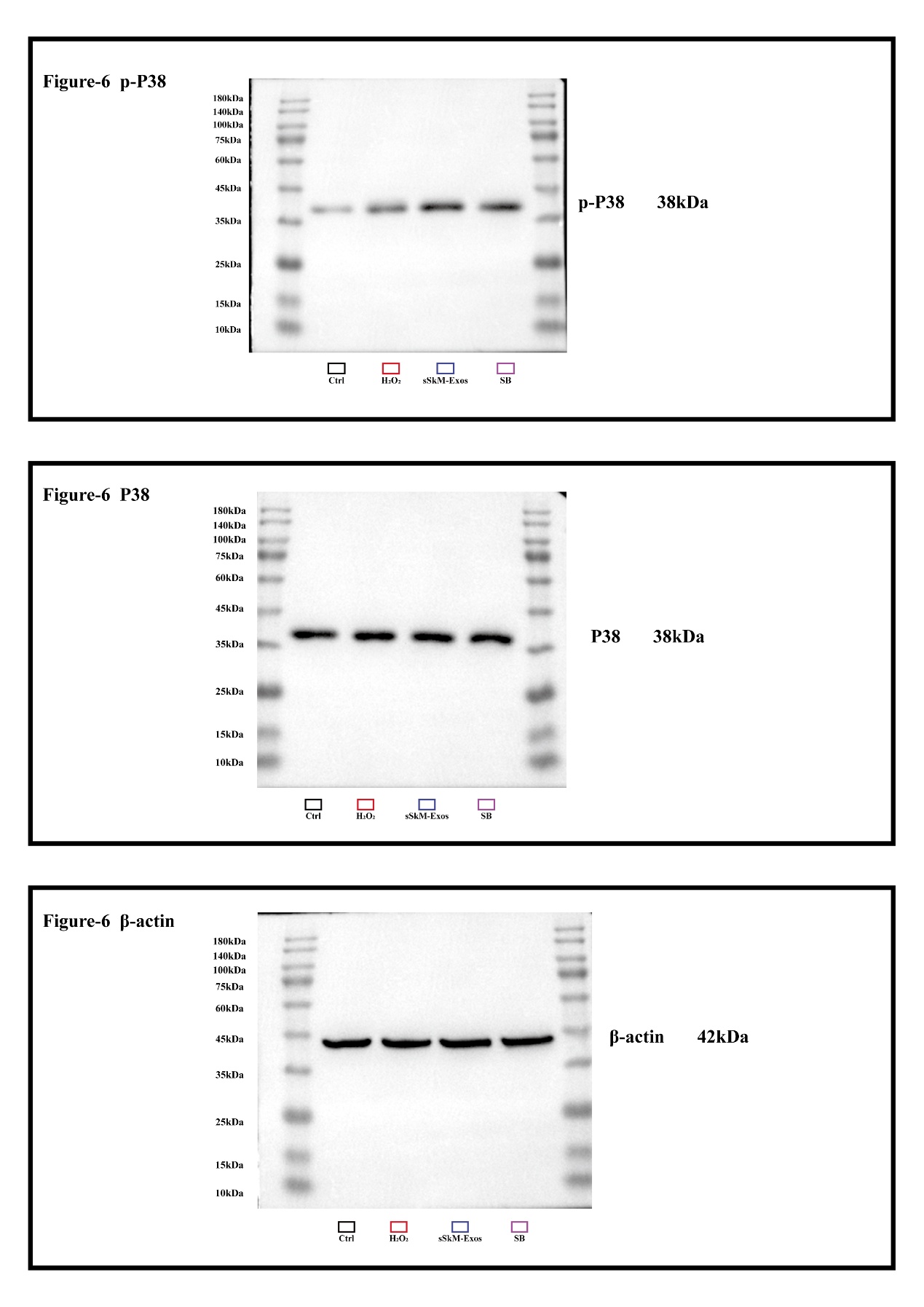

Supplement: Supplementary file 1 — Supplementary Material 1 [file 12891_2026_9816_MOESM1_ESM.docx]
